# Effects of Mechanical Force on Cytoskeleton Structure and Calpain-Induced Apoptosis in Rat Dorsal Root Ganglion Neurons In Vitro

**DOI:** 10.1371/journal.pone.0052183

**Published:** 2012-12-20

**Authors:** Zhengxu Ye, Yuqing Wang, Xin Quan, Jing Li, Xueyu Hu, Jinghui Huang, Zhuojing Luo

**Affiliations:** 1 Department of Orthopedics, Xijing Hospital, Fourth Military Medical University, Xi’an, Shaanxi, China; 2 Department of Pathology, Xijing Hospital, Fourth Military Medical University, Xi’an, Shaanxi, China; IISER-TVM, India

## Abstract

**Background:**

A sudden mechanical insult to the spinal cord is usually caused by changing pressure on the surface of the spinal cord. Most of these insults are mechanical force injuries, and their mechanism of injury to the spinal cord is largely unknown.

**Methods:**

Using a compression-driven instrument to simulate mechanical force, we applied mechanical pressure of 0.5 MPa to rat dorsal root ganglion (DRG) neurons for 10 min to investigate cytoskeletal alterations and calpain-induced apoptosis after the mechanical force injury.

**Results:**

The results indicated that mechanical forces affect the structure of the cytoskeleton and cell viability, induce early apoptosis, and affect the cell cycle of DRG neurons. In addition, the calpain inhibitor PD150606 reduced cytoskeletal degradation and the rate of apoptosis after mechanical force injury.

**Conclusion:**

Thus, calpain may play an important role in DRG neurons in the regulation of apoptosis and cytoskeletal alterations induced by mechanical force. Moreover, cytoskeletal alterations may be substantially involved in the mechanotransduction process in DRG neurons after mechanical injury and may be induced by activated calpain. To our knowledge, this is the first report to demonstrate a relationship between cytoskeletal degradation and apoptosis in DRG neurons.

## Introduction

Acute spinal cord injury (ASCI) is a major cause of persistent disability in humans [1], [2]. The pathological process of ASCI has two phases: the primary injury caused by a sudden mechanical force, and the secondary injury [3], [4]. The delayed, secondary spinal cord injury is considered to be due to a cascade of biochemical reactions that are important in the mechanism of ASCI [5]. In recent years, efforts have been made to clarify the apoptotic pathways of neuronal cells during the secondary injury. As a result, an increasing amount of evidence suggests that an increase in the intracellular calcium concentration may be the initial biochemical mediator in this process [6], [7]. Several cysteine proteases may also be involved [8], [9]. Caspases are the main cysteine proteases in apoptotic processes, and 14 caspases have been identified [10]. Except for caspases 1 and 11 [11], [12], the caspases are associated with apoptosis; however, caspase 3 appears to be the main player in neuronal cell apoptosis [13]. Furthermore, calpain, a calcium-dependent cysteine protease, is also involved in both the apoptotic and necrotic processes leading to neuronal cell death [14], [15], [16], [17]. Some studies have shown that calpain inhibitors provide neuroprotection during ASCI and indicate that calpain may have an important role in the resulting neuronal cell death [16], [17]. In addition, calpain has been proposed to work upstream of caspase 3 in the apoptotic process in rat SCI [18]. In the early stages of ASCI, increased levels of intracellular free calcium directly promote calpain activation [14]. Calpain subsequently degrades many cytoskeletal and membrane proteins in the neuron [19], [20], [21]. These cytoskeletal and membrane proteins provide architectural support for eukaryotic cells and are involved in mechanotransduction. Thus, activated calpain and increased intracellular free calcium levels after ASCI participate in neural apoptosis [22], [23].

Different chemical stimuli [24], [25] and mechanical injury [26], [27] induce apoptosis in various cell lines; however, the mechanism of action varies for different stimuli. For example, colchicine induces apoptosis in cerebellar granule neurons by degrading cytoskeletal structures [28], [29]. In this model, apoptosis is generally mediated through the release of cytochrome c and through caspase 3 activation [30]. In mechanical injury models, many researchers believe that the transduction of mechanical forces occurs through changes in protein conformation [31], [32], [33]. The cytoskeleton is important for sensing mechanical stimuli and has been demonstrated to increase cytoskeletal stiffness [34]. Unfortunately, the mechanisms involved in mechanical injury–induced apoptosis are poorly understood. It is not clear if cytoskeleton degradation is caused directly by mechanical forces or which cysteine proteases are involved in the apoptotic process. A better understanding of the apoptotic pathway in a cell model of mechanical injury may aid in developing more effective interventions for reducing the consequences of ASCI.

**Figure 1:**
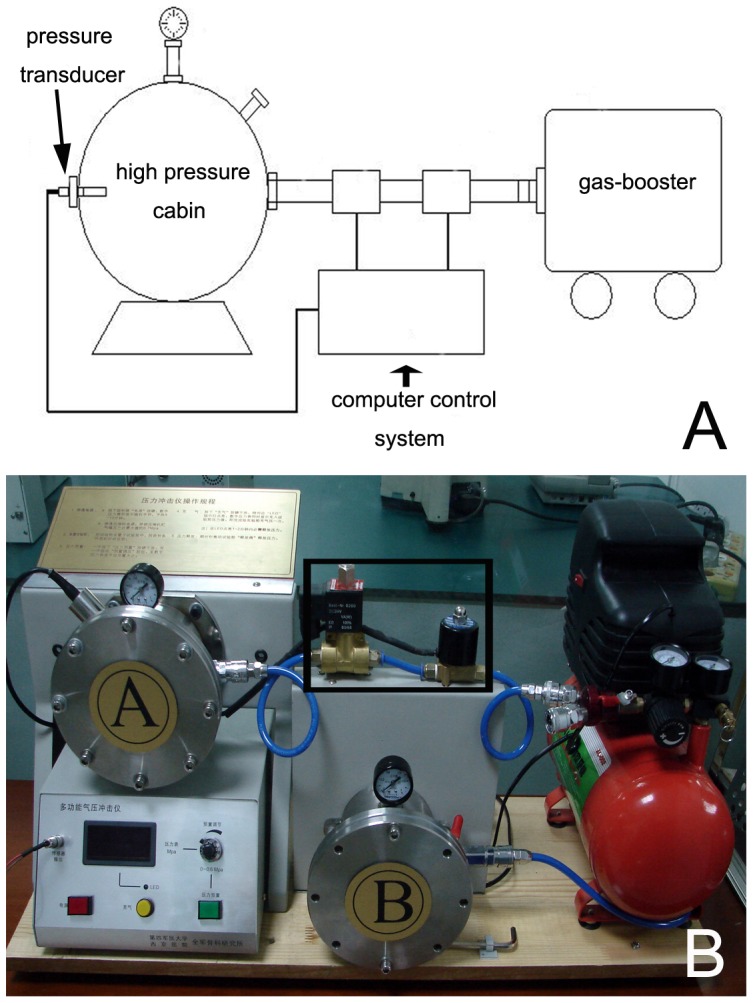
The device for applying pressure to cells. (A) A schematic of the custom-made instrument for applying mechanical pressure to DRG neurons. The main components of the instrument include a mechanical pressure container, a gas booster, and a pressure control system. (B) The mechanical pressure container (labeled “A”) is connected to a gas booster (red) with a sealed tube (blue). Two metal valve tubes (in black frame) are controlled by a computer system to regulate the air pressure.

Thus the aim of the present study was to investigate the underlying apoptotic pathways induced by mechanical force in dorsal root ganglion (DRG) neurons. We used a self-designed, mechanical pressure–controlled cellular injury unit to precisely generate 0.5 MPa of pressure on DRG neurons inside a high-pressure container, which was placed in a 37°C incubator for 10 min (Figure 1). We then evaluated the cytoskeletal alterations in DRG neurons and the effect of a highly specific inhibitor (PD150606) of calpain. We observed that calpain were involved in regulating apoptosis induced by mechanical force in DRG neurons. Moreover, cytoskeletal alterations, which may be important for the mechanotransduction process of DRG neurons after mechanical injury, may be induced by activated calpain.

## Results

### Effects of Mechanical Force on Cell Viability

**Figure 2:**
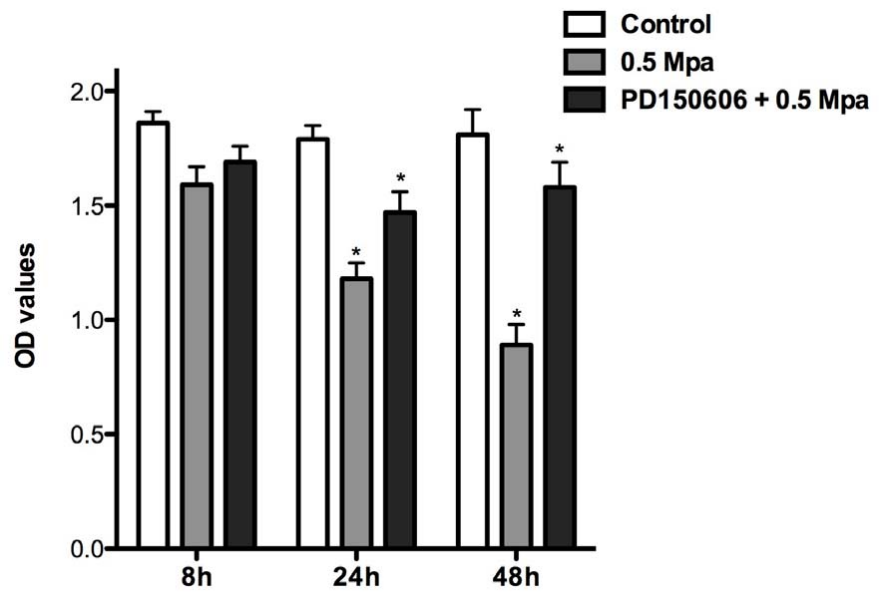
Effects of mechanical force on cell viability of DRG neurons. Compared with control cells, the OD values of DRG neurons treated with 0.5 MPa of pressure were significantly lower at 24 and 48 h after pressure treatment OR injury (P<0.05, n = 5). The OD values at 24 and 48 h after pressure treatment in cells treated with PD150606+0.5 MPa of pressure were significantly higher than those treated with 0.5 MPa of pressure alone (P<0.05, n = 5).

We first tested the hypothesis that mechanical injury influences the cell viability of DRG neurons and that calpain is involved this process. The results of the 3-(4,5-dimeth- ylthiazol-2-yl)-2,5 -diphenyltetrazolium bromide (MTT) assay are shown in Figure 2. The OD values of DRG neurons treated with 0.5 MPa of pressure were reduced in a time-dependent manner (P<0.05). Compared with control cells, the OD values of DRG neurons treated with 0.5 MPa of pressure were reduced in a time-dependent manner at 24 and 48 h after pressure treatment (P<0.05). The OD values in cells treated with PD150606+0.5 MPa of pressure were significantly higher than those in cells treated with 0.5 MPa alone at 24 and 48 h after pressure exposure (P<0.05).

**Figure 3:**
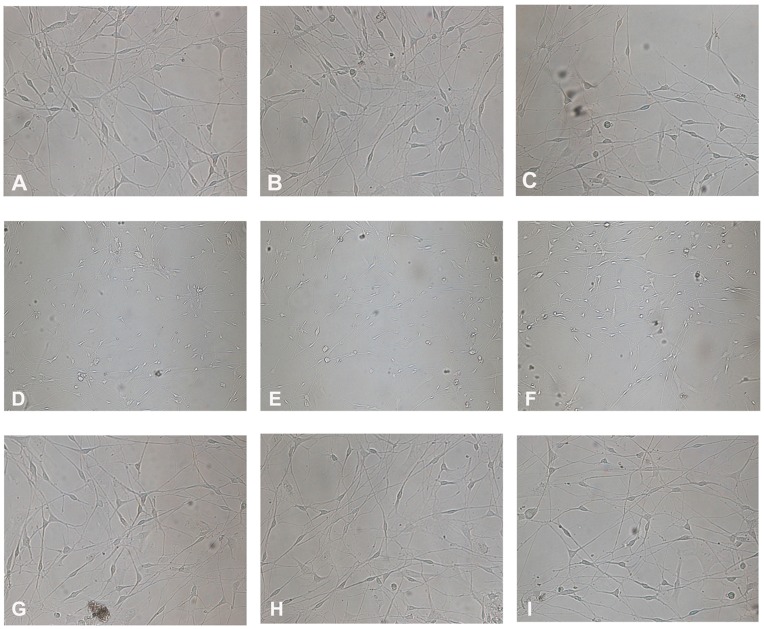
Effects of mechanical pressure on the morphology of DRG neurons. The sham control cells (A–C) displayed normal morphology. Cells subjected to 0.5 MPa of pressure for 30 min showed collapsed axons and other signs of necrosis after (D) 8 h, (E) 24 h, and (F) 48 h. Other cells were subjected to 0.5 MPa of pressure and treated with PD150606 for (G) 8 h, (H) 24 h, and (I) 48 h; these cells showed alleviation of pressure-induced morphological changes. All experiments were repeated at least three times.

Representative photographs of DRG neurons at each time point after injury are shown in Figure 3. Control cells displayed normal morphology and exhibited normal neurite outgrowth (Figure 3A–C). No apparent differences were observed between cells treated with 0.5 MPa of pressure and control cells 8 h after injury (Figure 3A, D). However, the morphology of cells exposed to 0.5 MPa of pressure was substantially different at 24 and 48 h after injury. Cells treated with 0.5 MPa of pressure exhibited more severe damage, and more floating cells were observed (Figure 3E–F). Cells treated with PD150606+0.5 MPa of pressure showed decreased cell density, and the neurites became shorter and irregularly shaped (Figure 3G–I). However, there was no significant difference between cells treated with 0.5 MPa of pressure and cells treated with PD150606+0.5 MPa of pressure at any time point.

### Effects of Mechanical Force on Cytoskeleton Structure

**Figure 4:**
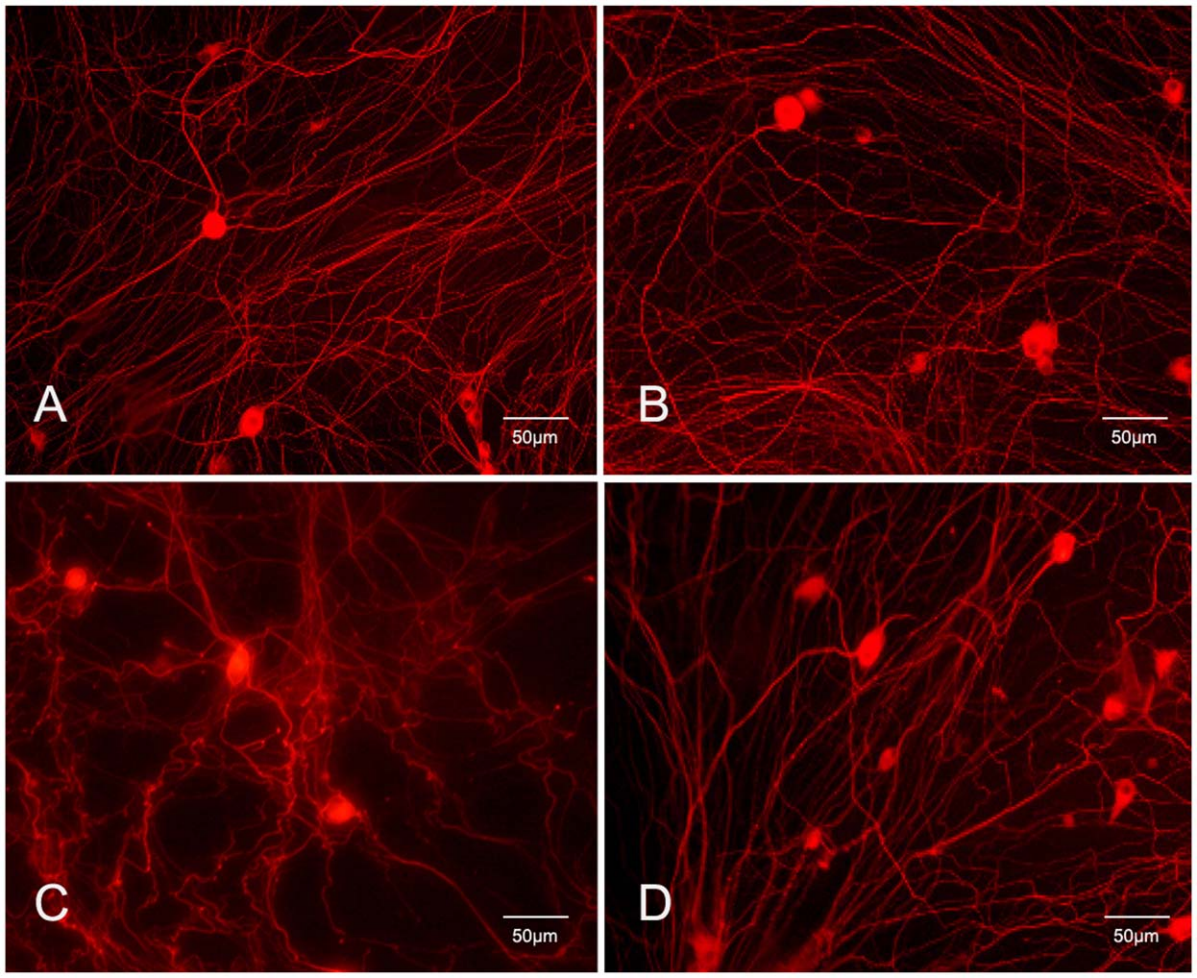
Effects of mechanical pressure on microtubules in DRG neurons βIII-tubulin. The expression of βIII-tubulin was mainly localized in the cytoplasm. There were no significant changes in control cells (A) or control+PD150606–treated cells over time (B). Cells subjected to 0.5 MPa of pressure for 30 min (C) showed retraction or collapse of neurites, coincident with a decrease in the expression of βIII-tubulin. However, in cells subjected to 0.5 MPa of pressure and treatment with PD150606 (D), the collapse of neurites as indicated by decreased βIII-tubulin was reduced, and βIII-tubulinexpression was significantly higher when compared with cells treated with 0.5 MPa of pressure without PD150606. All experiments were repeated at least three times.

Immunohistochemistry 48 h after injury verified the morphological observations. In control cells and cells treated with PD150606 only, staining for βIII-tubulin, a neuron-specific cytoskeleton protein, revealed an organized and continuous distribution of microtubules (Figure 4A–B). Microtubule disruption, disorganization, and some structural degradation were observed in the neurites of cells treated with 0.5 MPa of pressure (Figure 4C). However, in cells treated with PD150606+0.5 MPa of pressure, the cytoskeletal morphology indicated less damage, longer neurites, and a continuous distribution of microtubules (Figure 4D), which are indicative of the neuroprotective effects of PD150606.

### Effects of Mechanical Force on βIII-tubulin Expression

**Figure 5:**
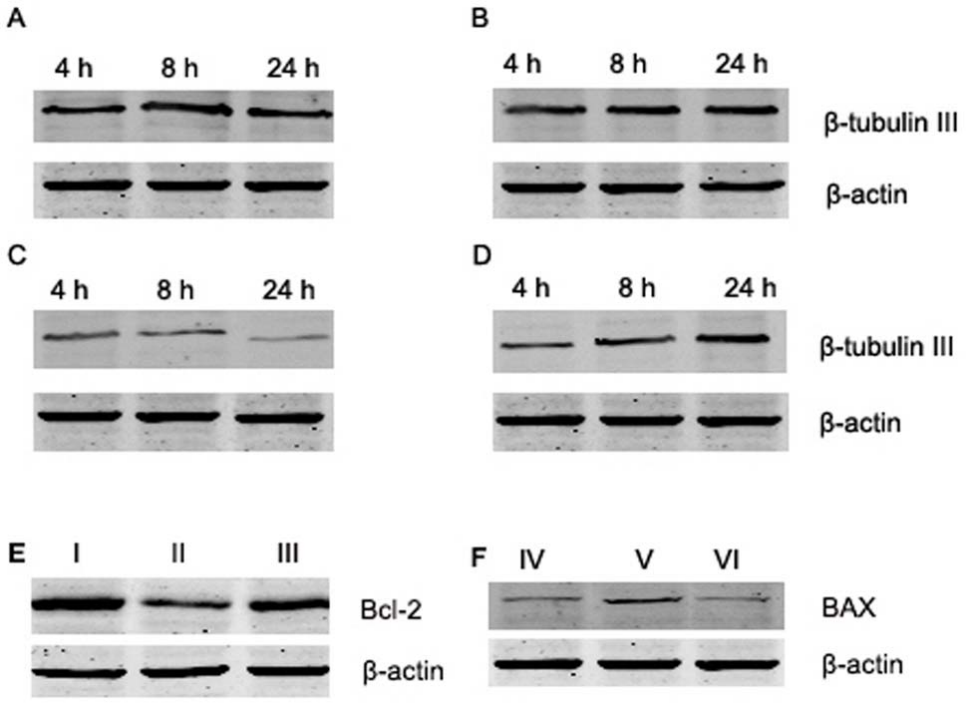
Changes in cytoskeletal and apoptosis-related protein expression in DRG neurons after exposure to mechanical pressure. After cells were subjected to mechanical pressure injury, the expression of βIII-tubulin III, Bcl-2, and BAX was determined with western blotting. The expression of βIII-tubulinis shown in (A) control cells, (B) control cells treated with PD150606, (C) cells treated with 0.5 MPa of pressure, and (D) cells treated with 0.5 MPa of pressure and PD150606. The expression of Bcl-2 is shown in (E): control cells (I), cells treated with 0.5 MPa of pressure (II), and cells treated with 0.5 MPa of pressure and PD150606 (III). The expression of BAX is shown in (F): control cells (IV), cells treated with 0.5 MPa of pressure (V), and cells treated with 0.5 MPa of pressure and PD150606 (VI). All experiments were repeated at least three times.

Western blotting also showed that the βIII-tubulin levels were similar at 8, 24, and 48 h after injury in control cells and cells treated with PD150606 only. βIII-tubulin levels in these cells were significantly higher than levels in cells treated with 0.5 MPa of pressure and with PD150606+0.5 MPa of pressure (P<0.05; Figure 5). However, the protein levels of βIII-tubulin in cells treated with PD150606+0.5 MPa were higher than levels in cells treated with 0.5 MPa of pressure alone at 8, 24, and 48 h after injury (P<0.05; Figure 5).

### Effects of Mechanical Force on Cell Apoptosis

**Figure 6:**
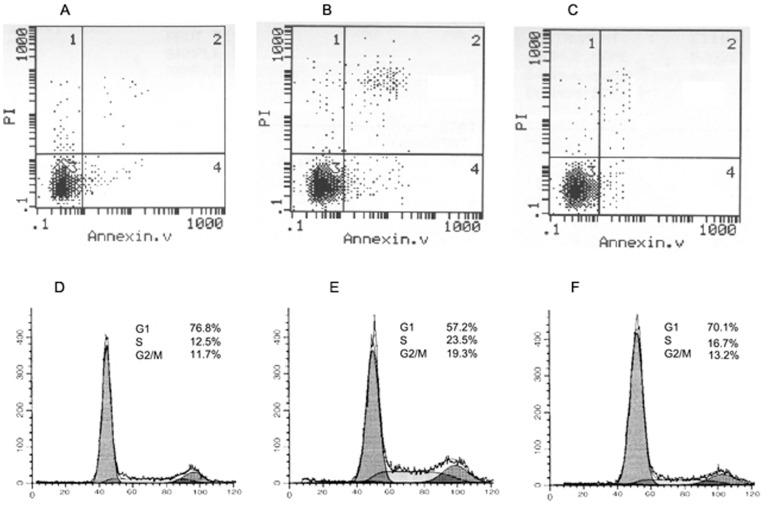
Cell cycle effects in DRG neurons after exposure to mechanical pressure. After cells were subjected to 0.1 MPa or 0.5 MPa of pressure, an increase in the number of apoptotic cells was observed 24 h after injury. Treatment with PD150606 significantly decreased the number of apoptotic cells in cells exposed to either 0.1 MPa or 0.5 MPa of pressure (P<0.05, n = 5). (A) control cells, (B) cells treated with 0.5 MPa of pressure, and (C) cells treated with 0.5 MPa of pressure and PD150606. Cell cycle distribution was examined 24 h after exposure to mechanical pressure in the different groups: (D) control, (E) cells treated with 0.5 MPa of pressure, (F) cells treated with 0.5 MPa of pressure and PD150606.

The number of apoptotic cells was counted and compared among the different groups of cells with flow cytometry analysis. The percentage of apoptotic cells in cells treated with PD150606+0.5 MPa of pressure was significantly lower than the percentage in cells treated with 0.5 MPa of pressure alone (P<0.05; Figure 6A–C). Cells that were treated with PD150606 and were exposed to mechanical pressure showed a decrease in the number of apoptotic cells as compared with the injury-only cells, suggesting that PD150606 had a neuroprotective effect. In addition, we also examined changes in apoptosis-related proteins with western blotting (Figure 5). In cells treated with 0.5 MPa of pressure, as compared with control cells, the expression of Bcl-2 was clearly decreased, whereas BAX expression was increased. In cells that were exposed to 0.5 MPa of pressure in the presence of PD150606, the level of Bcl-2 expression increased, as compared with the pressure-only cells, and expression of BAX also clearly decreased.

### Effects of Mechanical Force on the Cell Cycle

The cell cycle was investigated with flow cytometry in each treatment group. After mechanical injury with 0.5 MPa of pressure, the fraction of cells in S phase was increased compared with control cells (23.5% *vs.* 12.5%), and the fractions in G2/M phase were 19.3% and 11.7%, respectively (Figure 6D–F). Addition of PD150606 changed the cell cycle distribution of DRG neurons after mechanical injury. The fractions of cells in S phase and G2/M phase were 16.7% and 13.2%, respectively. This result indicates that blocking the calpain pathway by mechanical injury modulates the cell cycle distribution.

## Discussion

In the present study, we show for the first time the effects of mechanical force on cytoskeleton structure and calpain-induced apoptosis in DRG neurons in vitro. The primary findings are as follows: 1) mechanical force clearly affected the cytoskeleton structure and cell viability, induced apoptosis in DRG neurons, and affected the cell cycle; 2) calpain played a key role in this process, because blocking calpain with a specific inhibitor modulated the biological response of DRG neurons after exposure to mechanical force; and 3) the underlying mechanism of this process is related to βIII-tubulin, Bcl-2, and BAX. Cultured DRG neurons were subjected to mechanical injury with a custom-built compression system, and the resulting morphological changes and potential intracellular signaling cascades involved in apoptosis were investigated. To our knowledge, this is the first report to demonstrate a relationship between cytoskeletal degradation and apoptosis in DRG neurons.

Previous studies have demonstrated that calpain activation is involved in the pathophysiology of neurodegenerative disorders and CNS diseases [35], [36] and that calpain mediates apoptosis after SCI [15], [25]. Increased calpain expression and activation have also been detected in macrophages, reactive astrocytes, microglia, and neurons in SCI lesions [6], [37]. During SCI, an increase in intracellular calcium may be the first biochemical mediator and may stimulate calpain activity that mediates neuronal apoptosis. In addition, calpain activation is induced by increased intracellular free calcium within 15 min after the insult, with a peak in its activity by 2 h after injury [38]. Therefore, we treated DRG neurons with the calpain inhibitor PD150606 immediately after injury and examined its efficacy at 8, 24, and 48 h after the trauma. PD150606 produced a beneficial effect on morphological changes in this model.

The apoptotic process is regulated by cysteine aspartate proteases (caspases), but an increasing amount of evidence indicates that calpain is another important cysteine protease responsible for neuronal apoptosis. In addition, previous research has revealed that calpain activation is associated with the caspase cascade, leading to apoptosis after SCI. In a study of neonatal cerebral hypoxia-ischemia in rats, a direct link between early, calcium-mediated calpain activation and subsequent caspase-3 activation was revealed [39]. An association between calpain activity and caspase-12 cleavage has also been demonstrated in other studies [40]. Based on a previous report [18], the relationship between calpain and caspase-3 has been investigated in a rat SCI model, and the results suggest that calpain may act upstream of caspase-3 in mediating apoptosis after SCI. However, no reports have been published regarding other possible proteins involved in the downstream signaling pathway of calpain after mechanical injury, and, thus, changes in Bcl-2 and BAX were examined in our experiments. Bath application of the calpain inhibitor after mechanical injury in vitro not only counteracted the disruption of βIII-tubulin, a key protein of the neuronal cytoskeleton, but also reduced apoptosis. Our results demonstrated that calpain activation may play a major role in the cascade of mechanical injury–induced apoptosis in vitro.

In addition, administration of calpain inhibitors appears to aid in neuroprotection and functional recovery of CNS tissues. Schumacher et al. [41] demonstrated that CEP-4143, a μ-calpain inhibitor, significantly preserves tissue and improves behavioral outcomes in an animal model of experimentally induced SCI. Bartus et al. [42] showed that the calpain inhibitor AK 295, a tripeptidyl α-keto amide that strongly inhibits both forms (μ and m) of calpain in a reversible manner, can protect neurons from ischemia in the context of focal ischemic brain damage. In addition, many studies have suggested that AK 295 protects neurons and improves behavioral outcomes by preventing the degradation of cytoskeletal and membrane proteins, which are well-known calpain substrates. In contrast, PD151606, a selective calpain inhibitor, fails to prevent apoptosis mediated by colchicine in cerebellar granule neurons [43]. The most likely explanation is that in apoptosis induced by cytoskeletal alterations, selective inhibition of calpain is either not sufficient to prevent the apoptotic process or provides only partial neuroprotection against colchicine, delaying rather than preventing apoptosis.

In the present study, we demonstrate that the calpain inhibitor PD150606 allowed cells to maintain their cytoskeletal structure and provided neuroprotection against mechanical force–induced apoptosis. The effects of the calpain inhibitor may be achieved by preventing the degradation of cytoskeleton and membrane proteins. Therefore, we suggest that cytoskeleton degradation is not directly caused by the mechanical force in this model. The intracellular calcium increase may be the first biochemical event that follows mechanical force injury. However, the transduction of mechanical forces appears to occur through changes in certain membrane protein conformations, such as the sodium-calcium transporter, voltage-gated calcium channels, and calcium-permeable α-amino-3-hydroxy-5-methyl-4-isoxazolepropionic acid receptors, which play important roles in causing intracellular calcium overload following mechanical force injury [44]. Calpain are likely to be subsequently activated by increased intracellular calcium and participate in the degradation of the cytoskeleton and the initiation of apoptosis in DRG neurons.

Although DRG neuron death contributed to the excess calpain activation after mechanical force injury, other cysteine proteases (caspases) are likely to participate in the apoptotic process in this model. To elucidate the underlying apoptotic mechanisms after mechanical force injury, it will be critical to demonstrate an interaction between calpain and caspases. Therefore, future studies should investigate whether caspase inhibitors are effective in the neuroprotection of DRG neurons and whether calpain activation plays a crucial role in the determination of neuronal cell survival or death. This information is also important for physicians and surgeons when deciding whether a therapeutic strategy of calpain inhibitors after SCI will be effective and efficient.

In conclusion, our data provide strong evidence that calpain activation may play a major role in the cascade of mechanical force–induced apoptosis in vitro. The cytoskeletal alterations, which may function in the mechanotransduction process of DRG neurons after mechanical injury, may be induced by activated calpain.

## Materials and Methods

### Ethics Statement

All animal experiments were performed in accordance with the National Institutes of Health Guide for the Care and Use of Laboratory Animals and were approved by the Institutional Animal Ethics Committee of Fourth Military Medical University.

### Primary Cultures of DRG Neurons

Primary cultures of DRG neurons were prepared as described [45]. Briefly, newborn Sprague–Dawley rats were killed by decapitation and submerged in 95% ethanol for 2 min. DRGs were aseptically removed and saved along with as many rootlets as possible. The tissue was finely minced with eye scissors and digested in a mixture containing 0.1% collagenase and 0.25% trypsin for 25 min at 37°C. The cell suspension was then centrifuged at low speed for 4 min, and the supernatant was discarded. The DRG neurons were then gently washed three times for 5 min each in 5 ml DMEM with 10% FBS. The cell pellet was resuspended in neurobasal medium consisting of 1% B-27, 10 ng/mL nerve growth factor, 2500 mg/mL glucose, and 2 g/L NaHCO_3_. Cells were seeded onto coverslips or culture plates precoated with poly-l-lysine and laminin. Cultures were incubated at 37°C in a humidified atmosphere of 5% CO_2_/95% air, and the medium was replaced routinely every 2 days.

### Mechanical Pressure Injury to DRG Neurons and Experimental Design

We used a self-designed, mechanical pressure–controlled cellular injury unit, which was designed according to the model developed by Yousefian and Firouzian [46]. This unit consists of three parts: the mechanical pressure container, the pressure control system, and the gas booster (Figure 1). The unit can precisely simulate mechanical pressure injuries in vitro while excluding other confounding fact. In a preliminary experiment, cultured DRG neurons were treated with this mechanical device with 0.1, 0.5, and 0.7 MPa of pressure for 10 min, and cell viability was determined at 1, 8, 24, or 48 h with the MTT assay (Figure S1). Based on the preliminary results, mechanical pressure was set at 0.5 MPa for 10 min in the main experiment. The cultured DRG neurons were divided into four treatment groups as follows: control, 0.5 MPa of pressure, PD150606+0.5 MPa of pressure, and PD150606 alone. PD150606, a highly specific calpain inhibitor, was included in the medium to test the effects of calpain on DRG neuronal injury. All cells were cultured immediately after injury for 8, 24, or 48 h before subsequent experimental steps. The cells were first observed with an inverted phase contrast microscope at different times.

### MTT Assay

Cell viability was assessed at 8, 24, and 48 h after mechanical injury with the MTT assay [47]. DRG neurons were seeded at a density of 1 × 10^5^ cells/well in a 96-well plate after mechanical pressure injury. Then, 10 µl of 5 mg/ml MTT solution was added to the cells, and they were incubated at 37°C for an additional 4 h. The reaction was stopped by lysing the cells with 200 µl DMSO for 20 min. Absorbance was determined with a spectrometer at a wavelength of 570 nm. Wells without cells were used as blanks, and their values were considered background and subtracted from each sample.

### Immunohistochemistry Staining

Changes in βIII-tubulin were analyzed with immunohistochemistry. Cells from each treatment group that were grown on coverslips and then fixed with 4% paraformaldehyde in PBS for 25 min at room temperature, treated with 1% hydrogen peroxide for 10 min, and incubated for 40 min with blocking solution (1% BSA, 0.4% Triton X-100, and 4% normal serum in PBS). The cells were incubated with primary antibodies (1∶1000) overnight at 4°C and then incubated with FITC-conjugated rabbit anti–mouse IgG (1∶50) secondary antibodies at 37°C in the dark. Between each step, the cells were rinsed extensively three times for 5 min each. The stained cells were viewed with an Olympus fluorescent microscope by an observer blinded to the sample conditions.

### Western Blotting

The expression of βIII-tubulinand other related proteins was assessed with western blot analysis [48]. Briefly, at 8, 24, and 48 h after injury, DRG neurons in each group were harvested, centrifuged, lysed in 100 µl homogenization buffer (50 mM Tris; 150 mM NaCl; 5 mM EDTA, pH 7.5; 0.5% Triton X-100; 50 µM PMSF; 2 µg/ml aprotinin; 1 µg/ml pepstatin; and 1 µg/ml leupeptin), and denatured for 10 min at 100°C. Protein concentration of the cell lysate was measured using a commercial kit (Bio-Rad). Then, 40 µg protein per sample was separated with SDS-PAGE and transferred to a PVDF membrane (Millipore). The membranes were stained with washable Ponceau S solution to confirm equal protein loading. After washing, membranes were blocked in Tris-buffered saline with 0.02% Tween 20 and 5% nonfat milk. The proteins were then visualized using primary antibodies for βIII-tubulin, Bcl-2, and BAX (all at 1∶5000; Beyotime Biotech), followed by HRP-conjugated secondary antibodies (1∶1,000; Zymed, San Diego, CA). Specific proteins were detected using enhanced chemiluminescence (ECL; Amersham, Little Chalfont, UK), and the films were scanned. After washing with stripping buffer, the membrane was reprobed with antibody against β-actin (1∶2000). The band density of specific proteins was quantified using Quantity One 1-D Analysis Software (Bio-Rad) and normalized to the density of β-actin. Each western blot analysis was repeated two or three times with three to five samples in each group.

### Detection of Apoptotic Cells

The apoptosis rate of DRG neurons was detected and quantified with flow cytometry after staining with Annexin V–FITC and propidium iodide (PI; both from Roche, Mannheim, Germany). Stained DRG neurons in each group were harvested, washed, and suspended in Annexin V–FLUOS labeling solution. After a 15-min incubation at room temperature in the dark, the cell samples were analyzed with flow cytometry using a Becton Dickinson FACSCalibur (San Jose, CA, USA) using emission filters of 530/30 nm (FITC) and 585/42 nm (PI). A minimum of 10000 cells per sample was recorded, and cell debris was excluded with an appropriate forward light scatter threshold setting. The data obtained were analyzed with Cell Quest software (Becton Dickinson).

### Cell Cycle Analysis with Flow Cytometry

For cell cycle analysis, DRG neurons from each group were harvested with 0.125% trypsin and fixed with 75% (v/v) ethanol overnight at 4°C. The cells were then resuspended and incubated with 100 mg/µl RNase A at 37°C for 30 min. Cell nuclei were stained with 50 mg/ml PI for an additional 30 min. The distribution of cells in each phase of the cell cycle was determined with flow cytometry (Becton Dickinson), and DNA histograms were analyzed with Cell Quest software version 3.3 (Becton Dickinson). Each test was performed at least three times.

### Statistical Analyses

All values are expressed as the mean ± S.D. The statistical analyses were performed using SAS 6.04 software. A one-way ANOVA was used for multiple comparisons. The Student’s t-test was used to test whether the mean differed between two groups. The data were considered significant when the P-value was <0.05.

## Supporting Information

Figure S1 **Cell viability after different pressure of mechanical force.** In preliminary experiment, the cultured DRG neurons were performed with above mechanical device under 0.1, 0.5 and 0.7 MPa pressure for 10 min respectively, and cell viability were determined at 1, 8, 24 or 48 h by the methods of MTT.
(DOC)
