# Supplementary material for: Effects of Mechanical Force on Cytoskeleton Structure and Calpain-Induced Apoptosis in Rat Dorsal Root Ganglion Neurons In Vitro
Source: PLoS One. 2012 Dec 20;7(12):e52183. doi: 10.1371/journal.pone.0052183 (PMC3527405; doi:10.1371/journal.pone.0052183)
Supplement: Figure S1 — Cell viability after different pressure of mechanical force. In preliminary experiment, the cultured DRG neurons were performed with above mechanical device under 0.1, 0.5 and 0.7 MPa pressure for 10 min respectively, and cell viability were determined at 1, 8, 24 or 48 h by the methods of MTT. (DOC) [file pone.0052183.s001.doc]

**Supplementary data**


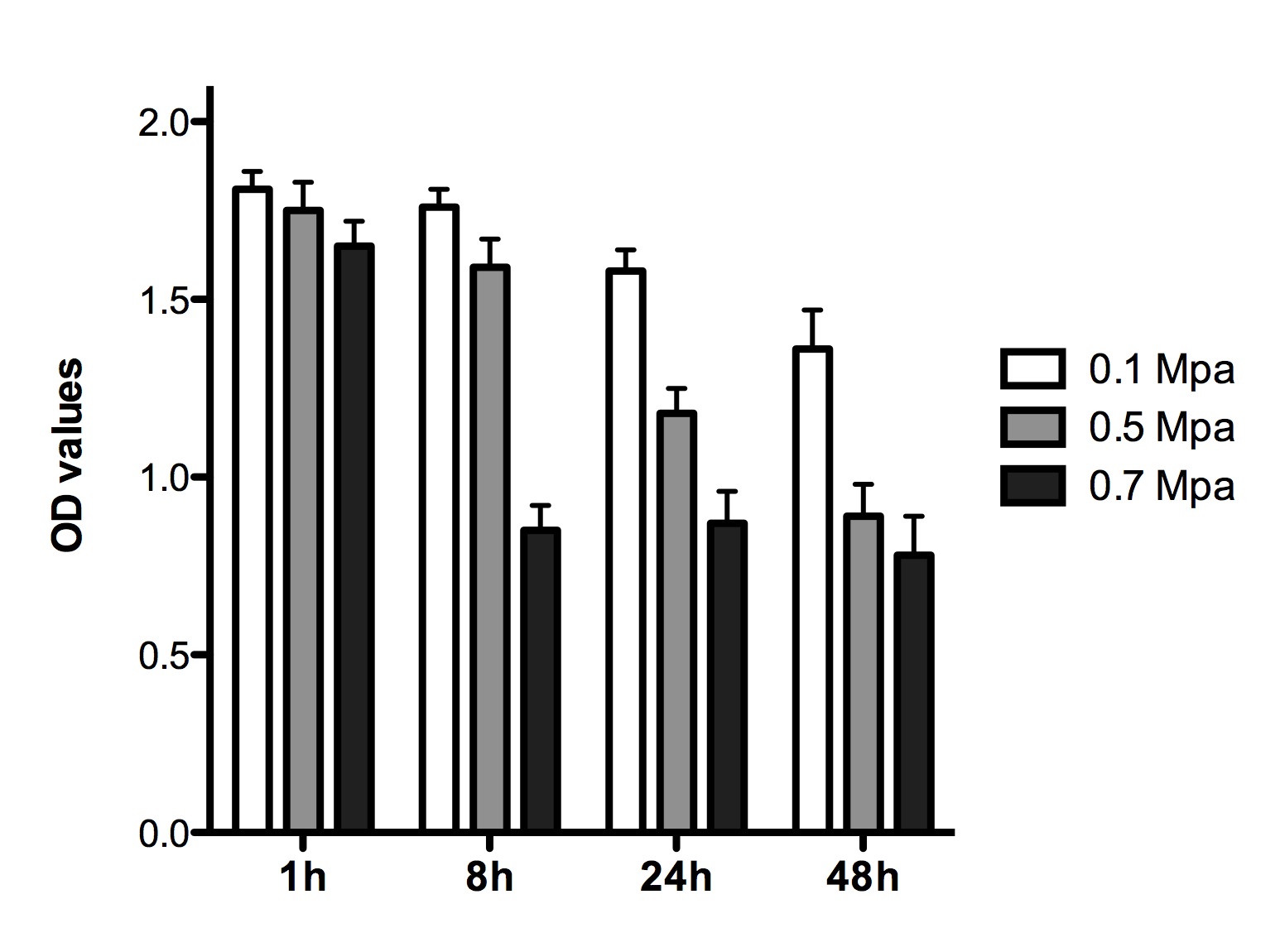


**Figure s1. Cell viability after different pressure of mechanical force.**

In preliminary experiment, the cultured DRG neurons were performed with above mechanical device under 0.1, 0.5 and 0.7 MPa pressure for 10 min respectively, and cell viability were determined at 1, 8, 24 or 48 h by the methods of MTT.
